# That song in my head: a review on Musical Hallucinations

**DOI:** 10.1192/j.eurpsy.2022.1826

**Published:** 2022-09-01

**Authors:** F. Ramalheira, M. Conde Moreno, J. Romão, S. Vieira

**Affiliations:** 1 Centro hospitalar Psiquiátrico de Lisboa, Serviço De Electroconvulsoterapia, Lisboa, Portugal; 2 Centro hospitalar Psiquiátrico de Lisboa, Hospital De Dia, Lisboa, Portugal; 3 Centro Hospitalar Universitário Lisboa Norte, Psychiatry, Lisboa, Portugal; 4 Centro Hospitalar Psiquiátrico de Lisboa, Psiquiatria Geral, Lisboa, Portugal

**Keywords:** Hallucination, musical, musical hallucinations, music

## Abstract

**Introduction:**

Hearing music inside our heads is frequent, however some hear it more vividly, constantly and involuntarily. Musical Hallucinations (MH), first described by Baillarger in 1846, are a complex type of auditory hallucination characterized by perception of melodies, music, or songs.

**Objectives:**

This work aims to review the literature considering MH.

**Methods:**

Pubmed and Google Scholar search using MeSH term “musical hallucinations”

**Results:**

MH occurs in 0.16% of the population. They´re usually perceived as frightening or annoying. Proposed mechanisms include spontaneous activity triggered by sensory deprivation from hearing impairment, like in visual hallucinations in Charles Bonnet syndrome, and some authors even include MH as a subtype of this syndrome. Indeed, 60% of all patients with MH have hearing impairment or deafness. Other less frequent causes include focal brain lesions involving the auditory pathway and cortex, temporal epilepsy, metabolic or drug intoxication. Psychiatric conditions are uncommon but not impossible, especially in affective disorders. MH most frequently consist in familiar tunes, sometimes of personal significance, religious songs (especially in older patients), childhood songs, folk and popular songs from the radio - suggesting that musical perception is never unlearned but represents a “parasitic memory”, an unchangeable memory feature which can be experienced by relevant neuronal circuit stimulation. Most patients with MH were reported to have no extraordinary musical skills.

**Conclusions:**

MH are rare and strongly associated with hearing loss, though investigation of other causes should be sought. Treating the underlying cause is important but remission is not guaranteed.

**Disclosure:**

No significant relationships.

